# Metal Oxide Nanoparticles: An Effective Tool to Modify the Functional Properties of Thermally Stable Polyimide Films

**DOI:** 10.3390/polym14132580

**Published:** 2022-06-25

**Authors:** Alexandra L. Nikolaeva, Alexander N. Bugrov, Maria P. Sokolova, Elena M. Ivan’kova, Ivan V. Abalov, Elena N. Vlasova, Iosif V. Gofman

**Affiliations:** 1 Institute of Macromolecular Compounds, Russian Academy of Sciences, 199004 St. Petersburg, Russia; anbugrov@etu.ru (A.N.B.); pmarip@mail.ru (M.P.S.); ivelen@mail.ru (E.M.I.); i.abalf@yandex.ru (I.V.A.); spectra@imc.macro.ru (E.N.V.); gofman@imc.macro.ru (I.V.G.); 2 Department of Physical Chemistry, Saint Petersburg Electrotechnical University (ETU “LETI”), ul. Professora Popova 5, 197022 St. Petersburg, Russia

**Keywords:** nanocomposites, polyimides, metal oxide nanoparticles, zirconia, titania

## Abstract

A series of polyimide/metal oxide (either ZrO_2_ or TiO_2_) nanocomposite films were fabricated based on two polymer matrices. The prepared films were characterized by scanning electron microscopy (SEM), atomic force microscopy (AFM), and X-ray diffraction analysis (XRD), and their thermal and mechanical properties were investigated with the use of thermogravimetric (TGA), differential thermal analysis (DTA), and thermomechanical analysis (TMA). We have found out that functional properties of the obtained materials are determined by a number of factors, not only the type, size, surface functionality, and concentration of the nanofiller, but also the chemical structure of the matrix polyimide. We have demonstrated some trends in the thermal and mechanical behavior of the materials depending on these features. The data could be of great interest in the areas where new materials with improved functional characteristics are needed.

## 1. Introduction

The development of modern technologies in a variety of industrial areas from medicine to aerospace necessitates working out new materials with either improved target characteristics or with an extended set of functional properties. Optimization of the design and performance of modern materials requires exploring the possibilities of controlling their physico-chemical characteristics. In the last 10–15 years, researchers from all over the world have focused mostly on the so-called nanocomposite materials, in particular on the polymer-inorganic ones [1,2,3,4,5], because of the relative easiness of application-oriented control of the functional properties of the resulting materials through the alteration of the physico-chemical characteristics of both constituents.

Aromatic polyimides (PIs) have long been used in many industrial applications due to their excellent mechanical and dielectric properties, corrosion resistance, high thermal and radiation resistance as well ass thermal stability. They are capable of replacing metals or glass in many areas such as electrical, mechanical, and aerospace engineering [6,7,8,9]. In recent years, the PI-based nanocomposite materials filled with metal oxide (MO) nanoparticles such as CeO_2_, ZrO_2_, NiO, TiO_2_, etc. have been extensively studied owing to the superiority of their functional properties over those of the unfilled PIs [10,11,12,13]. MO nanoparticles are now considered to form the basis of the diversity of modern “smart” materials and devices due to the possibility of manipulating their physical and chemical properties by changing the synthesis conditions of the nanoparticles and thus varying their characteristics such as size, morphology, surface stoichiometry (in particular, the number of oxygen vacancies), etc. [14,15]. As a result, the chemical activity of nanoparticles can change considerably, especially with regard to redox reactions and oxygen scavenging [16]. This feature of MO nanoparticles has predetermined their wide use in catalytic and sorption processes. However, the same features of the structure can play a key role in the thermal and especially thermo-oxidative stability of PI materials, providing variability of chemical interactions between the nanofiller and polymer matrix. We have already observed a quite intriguing effect of CeO_2_ nanoparticles on the thermal properties of PIs determined by the structure of the latter [10,17]. The introduction of nanosized ceria into the polymer matrix substantially increased the thermo-oxidative stability of only those PIs whose macromolecules contained SO_2_-groups, while that of a PI with no sulfonyl groups deteriorated by 30 °C. We suggested a mechanism of the interactions between the PIs and CeO_2_ nanoparticles which would explain such a selective impact basing on the hypothesis on the ability of the nanoparticles to take part in the formation of active oxygen species (AOS) on their surface. The AOS may well take part in a number of reactions between the nanoparticles’ surfaces and PI fragments affecting thermal properties of the polymers depending on their structure.

In this context, the capability of other transitional metal oxide nanoparticles to improve thermo-oxidative stability of PIs with various structures seems rather interesting, since these nanoparticles are known to possess quite active surfaces with regard to absorption of oxygen molecules from their surroundings and production of AOS [15]. Among the inorganic nanomaterials, TiO_2_ and ZrO_2_ are non-toxic, low-cost, and widely used nanofillers exhibiting high surface activity and good dispersibility in the matrix. They also possess high thermal and chemical stability, high refractive index, and dielectric constant. Moreover, they are able to crystallize in a number of polymorphs, whose structural parameters and physical properties differ from one another [18,19]. Both types of nanoparticles have shown good application prospects in improving the functional properties of PIs [12,13,20,21,22,23]. It should be pointed out that the electrical and optical properties of the PI-based nanocomposites doped with the said types of nanoparticles are discussed profoundly in the scientific literature, since the MO are known to possess a quite large band gap [22]. Data on the thermal and mechanical characteristics of the nanocomposites are scarcer and controversial, the same type of MO nanoparticles can affect these properties oppositely [13,21,24,25].

In this study, we have fabricated a set of nanocomposite materials doped with ZrO_2_ and TiO_2_. Since it is well-known that the surface activity of nanoparticles is determined by their chemical nature, size, and the presence of additional groups on the surface, we employed a number of synthesis techniques in order to investigate the impact of these factors along with the concentration of the nanofillers on the structure, morphology, and thermal and mechanical properties of aromatic PIs of various structures.

## 2. Materials and Methods

### 2.1. Materials

**Figure 1:**
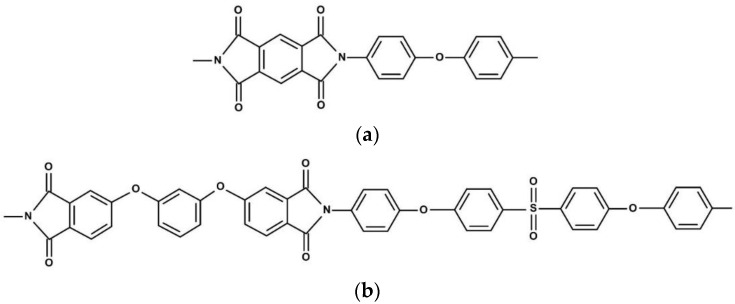
Structural formulas of the elementary units of the PIs: (**a**) PMDA-ODA; (**b**) R-BAPS.

Two thermally stable PIs were used both as matrices for nanocomposites and as reference bare materials: PMDA-ODA (PI with a repeating unit based on pyromellitic dianhydride (PMDA) and 4,4′-oxydianilin (ODA)) and R-BAPS (PI with a repeating unit based on 1,3-bis(3,4-dicarboxyphenoxy)benzene dianhydride (dianhydride R) and 4,4′-bis(4″-aminophenoxy)biphenyl sulfone (diamine BAPS)) (Figure 1). The polyamic acid (PAA) solution for preparation of PMDA-ODA-based samples was purchased from Sigma Aldrich (CAS: 25038-81-7, St. Louis, MI, USA). The PAA solution in N-methyl-2-pyrrolidone, NMP (Vekton, Saint Petersburg, Russia) for R-BAPS was synthesized in the Institute of Macromolecular Compounds of Russian Academy of Sciences (IMC RAS). Dianhydride R was provided by Tech. Chim. Prom. Ltd., Yaroslavl, Russia, diamine BAPS was purchased from TCI (>98%, CAS: 13080-89-2, Tokyo, Japan).

Zirconyl chloride octahydrate ZrOCl_2_·8H_2_O (98.5%, CAS: 7699-43-6) was provided by Neva-Reactive (Saint Petersburg, Russia). Zirconium acetylacetonate Zr(C_2_H_7_O_2_)_4_ (97%, CAS: 17501-44-9) and toluene (99.5%, CAS: 108-88-3) were provided by Vekton (Saint Petersburg, Russia). Titanium butoxide (purum, ≥97.0%, CAS: 5593-70-4) was purchased from Sigma Aldrich (St. Louis, MI, USA). Ammonia solution NH_4_OH (25%, CAS: 1336-21-6) was purchased from Vekton (Saint Petersburg, Russia) and used for the precipitation of zirconium oxyhydroxide as a precursor for the synthesis of nanoparticles. Ethyl alcohol (purity 98%, CAS: 64-17-5) provided from Vekton (Saint Petersburg, Russia) was used to wash nanoparticles from organic residues of precursors thermally decomposed during the synthesis.

### 2.2. Synthesis of Nanoparticles and R-BAPS Matrix Polymer

To assess the effect of the preparation method of the nanoparticles on the properties on the nanocomposite materials, zirconium dioxide powders were synthesized from precursors of various natures. In the case of using inorganic salt ZrOCl_2_·*8H_2_O, the precipitation of zirconium oxyhydroxide was carried out with the use of an aqueous solution of ammonia, followed by washing the formed precipitate by decantation. The precipitate was dried at 100 °C to constant weight and then subjected to hydrothermal treatment. A four-h isothermal treatment of ZrO(OH)_2_ under hydrothermal conditions at 250 °C and a pressure of 15 MPa led to the formation of nanocrystalline zirconium dioxide with a large number of hydroxyl groups remaining on their surface (Figure S1) [26]. The resulting powder is designated ZrO_2(hydroph)_. In some cases, ZrO_2(hydroph)_ nanoparticles were dried at 160 °C for 1 h in order to remove the hydroxyl groups from the surface, the obtained powder being designated ZrO_2(dried)_.

An alternative approach was the high-temperature hydrolysis of organic Zr(C_2_H_7_O_2_)_4_ under solvothermal conditions [27]. The resulting suspension of nanoparticles was washed with ethanol while stirring and centrifuging, and then dried to a constant weight. To remove residual organic impurities from the surface of nanoparticles and to control their size, zirconia powders were annealed in air at 500 °C for 2 h, and at 800 °C for 10 min and 2 h according to the temperature-time dependencies of their sizes presented in [28]. Nanoparticles of sizes 8, 18, and 28 nm were obtained, and designated ZrO_2_(8 nm), ZrO_2_(18 nm), ZrO_2_(28 nm), respectively.

Titanium dioxide nanoparticles were obtained by solvothermal synthesis. Titanium butoxide was added dropwise to toluene and stirred continuously for 24 h, the reaction mixture was then transferred to a steel autoclave with a Teflon cell with 80% filling factor. The parameters of isothermal treatment were chosen in accordance with the method presented in [29], the TiO_2_ nanoparticles obtained being of size 14 nm.

In order to synthesize R-BAPS polyimide the diamine BAPS was dissolved in NMP at 20 °C under argon. Then, an equimolar amount of dianhydride R was added to the resulting solution while stirring. The reaction was carried out in an argon flow. Stirring was continued for five hours to complete the polycondensation process.

### 2.3. Preparation of Pristine and Nanocomposite Films

PI/MO nanocomposites were fabricated by a standard solution casting technique [30,31]. The proper amounts of MO nanoparticles in NMP were sonicated for 1 h and then mixed with corresponding PAA solutions. The nanocomposite solutions were stirred for 24 h in order to form a quasi-homogeneous system. The compositions obtained as well as the reference bare PAA solutions were cast on glass supports and dried for 4 h at 80 °C. The final processing of the films obtained was performed by gradual heating up to 365 °C for PMDA-ODA and 300 °C for R-BAPS, followed by curing at these temperatures for 30 min. A list of the prepared nanocomposite samples is presented in Supplementary (Table S1).

### 2.4. Characterization Techniques

In order to investigate the structural and morphological features of the nanocomposite samples obtained as well as their constituents, a number of techniques were used.

Scanning electron microscopy (SEM) was performed on the cryo-cleavages of unfilled and nanocomposite films using a SUPRA-55VP microscope (Carl Zeiss, Oberkochen, Germany) equipped with a secondary electron detector. The specimens were fixed on microscope holders with special glue and covered with a thin layer of platinum.

Atomic force microscopy (AFM) data were obtained using an SPM-9700HT scanning probe microscope (Shimadzu, Kyoto, Japan). The AFM images were taken in air at room temperature. For the instrument operation, we chose a tapping mode, using NCHR-30SS Silicon tips with curvature radius less than 2 nm. The images 1024 × 1024 points were obtained.

IR spectra of unfilled and nanocomposite films were recorded on a Vertex 70 IR Fourier spectrometer (Bruker, Billerica, MA, USA) with the ATR (Attenuated Total Reflection) reflector (Pike Technologies, WI, USA) at room temperature in the range of 4000–500 cm^–1^ (resolution 4 cm^–1^, number of scans 30) with a ZnSe working element. When registering the ATR spectra, a correction was introduced that takes into account the penetration depth depending on the wavelength. The equipment was employed to confirm the presence of functional groups on the surface of ZrO_2(hydroph)_ nanoparticles.

X-ray phase analysis of the samples was carried out on a Rigaku SmartLab 3 diffractometer (Rigaku Corporation, Tokyo, Japan) with CuKα radiation. The diffraction (XRD) patterns were taken in the range of angles 2θ = 10–60° at a speed of 1°/min. The phase composition of the nanoparticles was determined with the use of PDWin 4.0 software (NPO “Burevestnik”, Russia) employing profile analysis of XRD patterns, the results of the analysis being compared with the ASTM database. The crystallite size was calculated from the broadening of X-ray diffraction lines according to the Scherrer equation [32].

Thermal properties of the materials were investigated with the use of a DTG-60 setup (Shimadzu, Kyoto, Japan) capable of simultaneous thermogravimetric (TGA) and differential thermal analysis (DTA). The specimens were heated up to 600 °C at a rate of 5 °C/min in air flow (80 mL/min). Thermal characteristics τ_10_ and τ_50_ (the temperatures at which a polymer or a composite loses 10 and 50% of its initial weight, respectively, because of thermal destruction) were determined using TGA curves. Data on glass temperature, *T*_g_, of the samples as well as on their supramolecular structure were obtained from DTA curves.

A thermomechanical analysis (TMA) was employed in order to investigate the mechanical behavior of the film samples under heating. A TMA 402 F1 Hyperion thermal Analyzer (NETZSCH, Selb, Germany) was used. The specimens were heated at a rate of 5 °C/min in argon flow (70 mL/min). *T*_g_ values were determined from TMA curves.

The study on the mechanical characteristics of the unfilled and nanocomposite PI films was conducted with the use of an AGS-X 5kN (Shimadzu, Kyoto, Japan) setup. Mechanical tests were carried out in a uniaxial extension mode at room temperature. Strip-like samples 2 × 30 mm^2^ in size were stretched at a rate 10 mm/min. The Young’s modulus E, yield stress σ_y_, break stress σ_b_, and the ultimate deformation ε_b_ for each sample were determined. For each composition 8–10 strips were tested, and the average and the standard deviation were calculated.

## 3. Results

### 3.1. Investigation of Structure and Morphology

**Figure 2:**
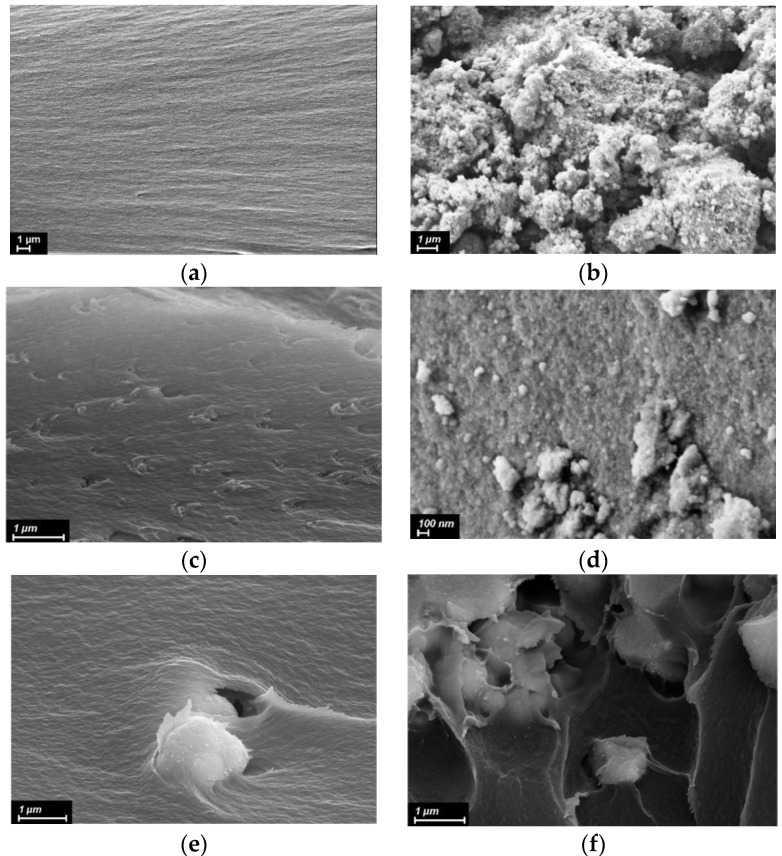
SEM images of (**a**) pristine PMDA-ODA matrix; (**b**) pristine TiO_2_ powder; (**c**) PMDA-ODA/5% TiO_2_; (**d**) pristine ZrO_2_(8 nm) powder; (**e**) PMDA-ODA/3%ZrO_2_(8 nm); (**f**) PMDA-ODA/7% ZrO_2_(8 nm).

**Figure 3:**
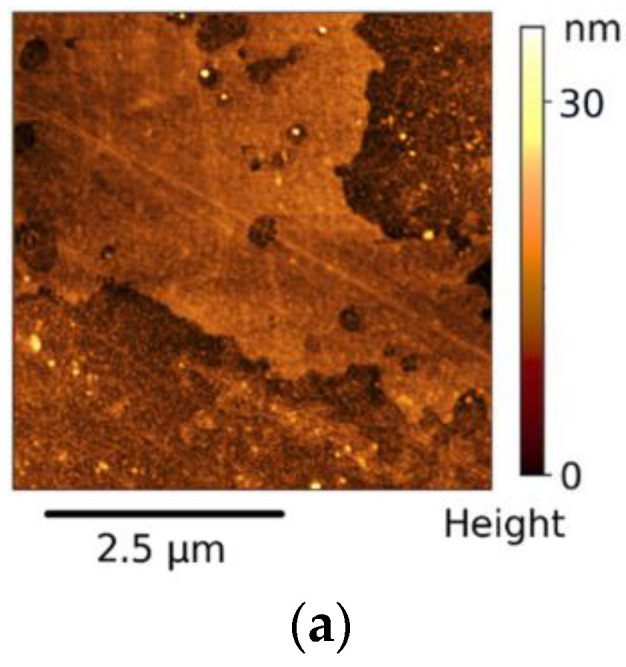

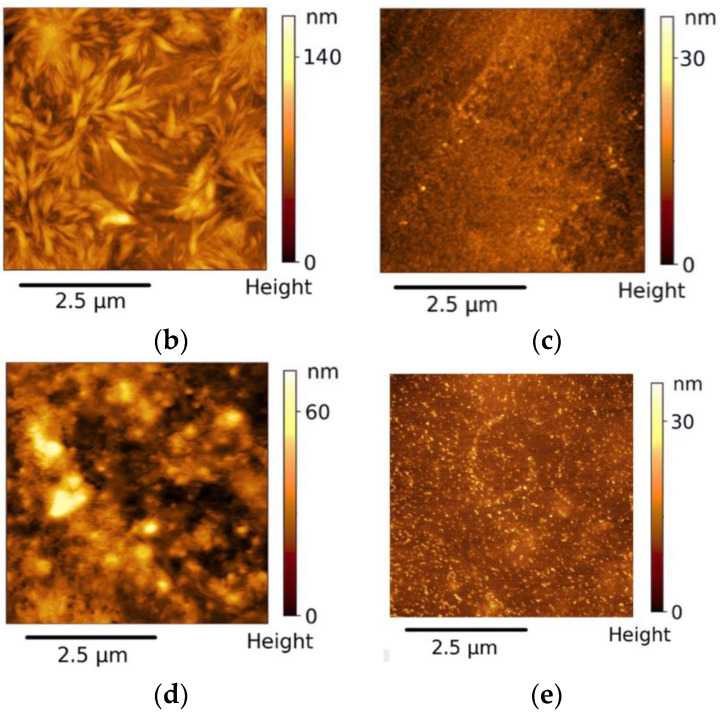
AFM images of initial PMDA-ODA film (**a**) and films with different oxide particles: (**b**) PMDA-ODA/5%ZrO_2_(8 nm); (**c**) PMDA-ODA/5%ZrO_2_(18 nm); (**d**) PMDA-ODA/5%ZrO_2(hydroph)_; and (**e**) PMDA-ODA/5%ZrO_2(dried)_.

To assess the distribution of the nanofillers within polymer matrices and to investigate the morphology of the materials obtained, SEM and AFM images of the samples were analyzed (Figure 2 and Figure 3).

Figure 2 shows SEM images of the cross-sections of polymer (bare and nanocomposite) films as well as of the pristine zirconia and titania powders. Smooth dense packing structure was observed in a SEM image of the bare PMDA-ODA (Figure 2a). The effect of the nature and concentration of MO nanoparticles on the morphology of PI was investigated. Multiple fracture lines (traces of plastic deformation) are seen on the cross-section of the nanocomposite containing 5 wt.% of TiO_2_ nanoparticles, aggregates of ca. 100–200 nm in size being homogenously dispersed in the PI matrix (Figure 2c). In contrast, ZrO_2_(8 nm) tends to form quite large (up to 1 µm) aggregates in the matrix. The distribution of these aggregates within the PI matrix was still uniform and the polymer seemed to get adhered to their surface (Figure 2e). The morphology of the cross-section was observed to be smooth. An increase in ZrO_2_(8 nm) concentration up to 7 wt.% led to a drastic change in the morphology of the nanocomposite. Large zirconia aggregates embedded in the cavities were detected (Figure 2f), implying their poor compatibility with the polymer matrix and nonuniform distribution of the nanofiller.

The AFM was used to detect changes in the morphology and roughness of the nanocomposite PMDA-ODA films depending on the size and surface functionality of the MO nanoparticles. Analysis of AFM images of the initial PMDA-ODA film (Figure 3a) shows a quite uniform morphology with low root mean square roughness Rq = 1.6 nm. The AFM images of the surface topography of the nanocomposite films (Figure 3b–e) demonstrate that the morphology is strongly dependent on the type of MO nanoparticles. Introduction of 5 wt.% of ZrO_2_(8 nm) into the PI led to a significant increase of the surface roughness of the films up to Rq = 13.5 nm (Figure 3b), while the incorporation of the same amount of ZrO_2_(18 nm) changed this characteristic only slightly, Rq = 2.0 nm (Figure 3c).

Comparing Figure 3d,e we also found that drying of the as-prepared zirconia powder had a direct impact on the surface topography and roughness of the material. Introduction of ZrO_2(hydroph)_ caused the formation of clearly visible nodular topography (Figure 3d) while the surface of the PMDA-ODA/5%ZrO_2(dried)_ film demonstrated smooth morphology with the emergence of uniformly distributed hills (Figure 3e), probably clusters of ZrO_2_. The values of surface roughness were estimated to be Rq = 9.0 nm and 2.0 nm for PMDA-ODA/5%ZrO_2(hydroph)_ and PMDA-ODA/5%ZrO_2(dried)_ samples, respectively.

**Figure 4:**
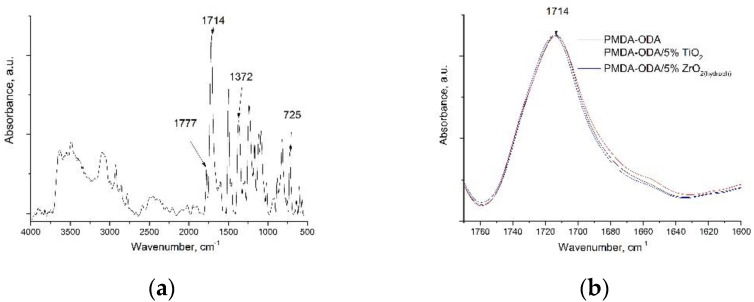
IR spectra of film samples: (**a**) initial PMDA-ODA, (**b**) PMDA-ODA composites with various types of nanoparticles.

Figure 4 shows IR spectra of the bare and nanocomposite PI film samples. The spectrum of the initial PMDA-ODA film exhibited the characteristic peaks at 1777 cm^−1^ (symmetric C=O stretching vibrations), 1714 cm^−1^ (antisymmetric C=O stretching vibrations), 1372 cm^−1^ (C–N stretching vibrations), and 725 cm^−1^ (deformation vibrations of C=O in the cycle), attributed to the imide cycle (Figure 4a), which indicates the conversion of PAA to the corresponding PI. When zirconium dioxide nanoparticles were introduced into the PI matrix, a change in the shape of the band of antisymmetric stretching vibrations of the C=O group of the imide ring was observed (Figure 4b). A slight shift to the high-frequency region, which is a typical sign of the weakening of the hydrogen bond network, took place. In contrast, the noticeable broadening of the same band to the low-frequency region was recorded for a PMDA-ODA filled with TiO_2_ nanoparticles, which may indicate the development of a network of hydrogen bonds.

**Figure 5:**
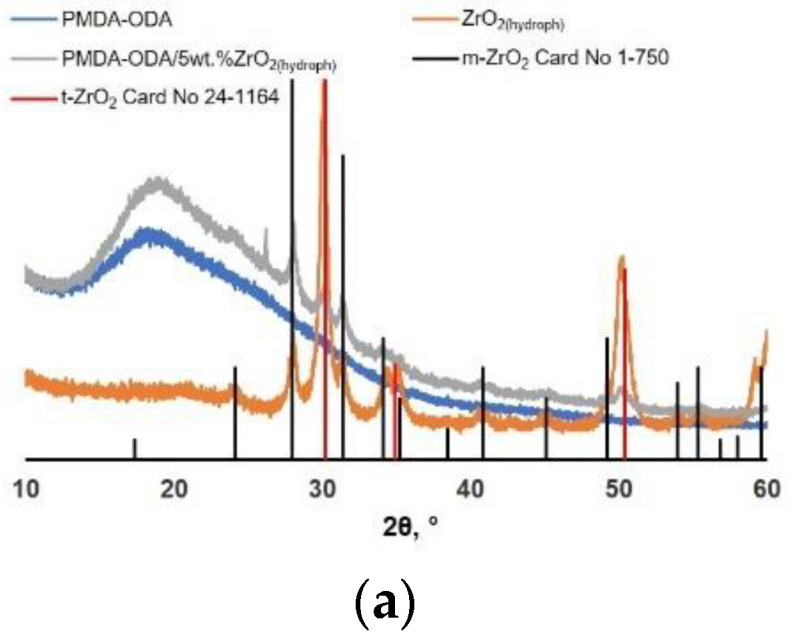

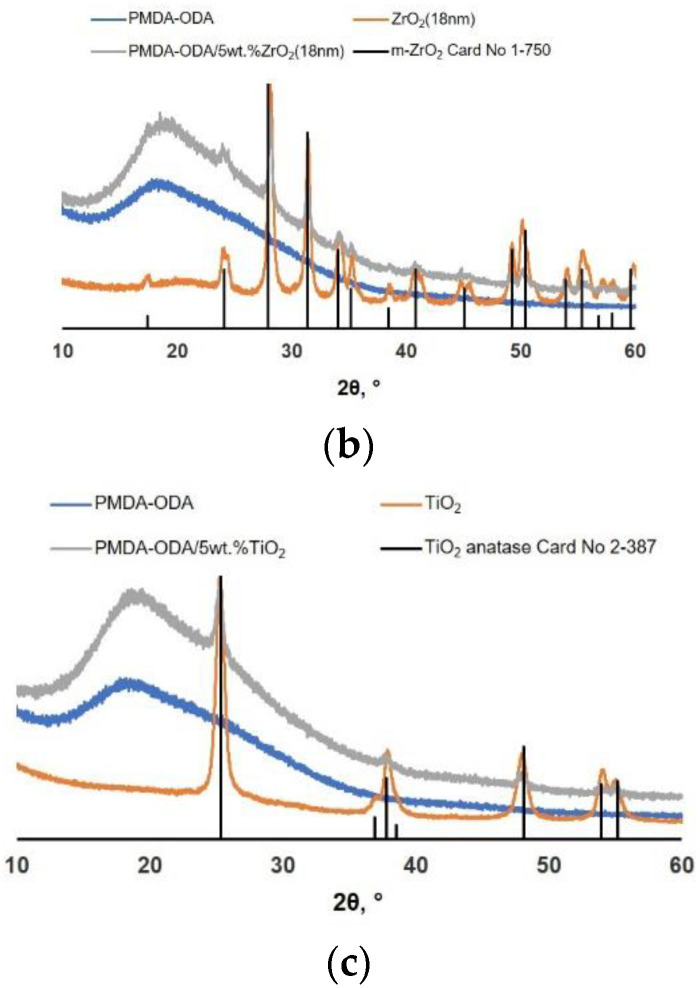
X-ray diffraction patterns of nanocomposite samples: (**a**) PMDA-ODA/ZrO_2(hydroph)_; (**b**) PMDA-ODA/ZrO_2_(18 nm); (**c**) PMDA-ODA/TiO_2_. The XRD patterns of the initial PMDA-ODA as well as of the corresponding MO nanoparticles are also presented. Quantitative phase analysis of XRD pattern of TiO_2_ nanoparticles (Figure 5c) using the ASTM database indicates that the nanoparticles possess the anatase crystalline structure. The average crystallite size was calculated using the Scherrer formula and equaled 14 ± 2 nm.

According to X-ray diffraction data, a powder of zirconium dioxide nanoparticles obtained from an inorganic precursor consisted of a mixture of tetragonal and monoclinic polymorphs in a ratio of 80:20 (Figure 5a). The crystallite size of the monoclinic phase was 21 ± 3 nm, and that of the tetragonal phase was 17 ± 2 nm. In the case of using the organic chelate-forming compound zirconium acetylacetonate as a precursor in the synthesis of nanoparticles, only crystallites in the monoclinic modification were formed. The size of m-ZrO_2_ crystallites was assessed to be 8 ± 1 nm. Subsequent annealing of the powder at of 800 °C for 10 and 120 min resulted in the removal of the residues of organic compounds from the surface of nanoparticles and led to an increase in the average size of the coherent scattering region (CSR) up to 18 ± 2 (Figure 5b) and 28 ± 2 nm, respectively.

A broad amorphous halo was registered on the XRD pattern of the PMDA-ODA film in the region of diffraction angles of 13–40° (Figure 5a–c, blue curve). In the case of composite films, one can also observe reflections corresponding to the initial MO nanoparticles which the PI was filled with (Figure 3 and Figure 4, blue, gray, and orange curves). The shape and position of PMDA-ODA halo remained intact implying that the amorphous structure of the PI did not change by MO nanoparticles.

### 3.2. Thermal Properties

**Figure 6:**
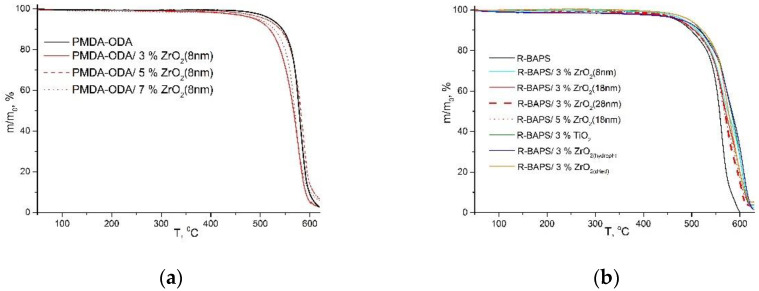
Thermogravimetric curves of (**a**) PMDA-ODA-based samples and (**b**) R-BAPS-based samples.

Figure 6 and Table 1 demonstrate the effect of the MO nanofillers on the thermal properties of PI-based nanocomposites. All the TGA curves revealed excellent thermal stability of the samples up to ~420 °C. At the first stage (up to 300 °C), all the residues of low-molecular weight compounds and surface water were removed from the samples, the weight loss at this step being ca. 2 wt.%. This implies the complete imidization of PAAs and the formation of PIs during the film fabrication. The TGA curves of all the investigated samples showed a single degradation step. However, the impact of the MO nanoparticles on the thermal behavior of the nanocomposites differs depending on the PI matrix used. A PI containing sulfonyl groups (R-BAPS) has its thermal stability enhanced being filled with MO nanoparticles. On the other hand, one may notice contradictory trends in the thermal behavior of a PI with no SO_2_-groups (PMDA-ODA) doped with these nanoparticles depending on their characteristics. Such a discrepancy between the two matrices agrees well with the data we reported earlier [10,17] for another type of MO nanoparticle, namely CeO_2_. The mechanism of PI-MO nanofiller interactions causing the enhancement/deterioration of PIs’ thermal stability is discussed in [17]. In brief, active oxygen species (oxide, peroxide, superoxide) (AOS) formed on the surfaces of MO nanoparticles during PI heating in air are supposed to take part in a number of chemical processes, inducing thermo-oxidative decomposition of the nanocomposite material.

It is well known that the smaller the nanoparticles, the more defects responsible for adsorption of molecular oxygen and formation of the AOS are present on their surface [15]. It is obvious from Table 1 that addition of ZrO_2_ nanoparticles of 8 nm in size in PMDA-ODA leads to a noticeable decrease in thermal stability of the polymer, while that of PMDA-ODA-based nanocomposites containing larger nanoparticles (18 and 28 nm) is even higher compared to the pristine matrix. Taking into account the AFM data (Figure 3c), demonstrating a more homogeneous surface topography of the sample containing ZrO_2_(18 nm), and enhanced mechanical properties of this nanocomposite (discussed below in Section 3.3), one would suggest that larger nanoparticles are less prone to aggregation and they facilitate the formation of additional links between PI macromolecules hindering the diffusion of free radicals and retarding the degradation of the nanocomposites.

An increase in ZrO_2_(8 nm) concentration up to 5 wt.% causes a substantial increase in thermal stability (see Figure 6a and Table 1) which likely results from the agglomeration of the nanoparticles with high surface energy [33] and the reduction in the number of the centers producing the AOS. The agglomeration is also confirmed by SEM images (compare Figure 2e,f) as well as by the gradual deterioration in mechanical properties of the nanocomposites (discussed below in Section 3.3) with higher contents of nanoparticles, since it is well known that the aggregates cause inhomogeneity of a resulting material reducing its mechanical strength [2,34]

In the case of R-BAPS possessing SO_2_-groups in its macromolecules, we presumed the following mechanism of PI-nanofiller interactions [17]. Sulfonyl radicals (probably formed at the first step of the PI’s thermal decomposition) bearing a positive charge on a sulfur atom can be electrostatically attached to the MO nanoparticles with negatively charged oxygen species adsorbed on their surfaces, causing a decrease in the number of volatile products in a certain temperature interval and thereby improving thermal stability of the R-BAPS-based nanocomposites (see Figure 6b and Table 1). One should notice that the enhancement was observed regardless of the type, preparation method, size, and concentration (at least in the investigated range) of the nanoparticles.

Analyzing the effect of zirconia preparation technique on the thermal stability, one can observe no substantial difference in the trends in the thermal behavior of the nanocomposites fabricated with the use of either zirconyl chloride or zirconium acetylacetonate as a precursor for nanoparticles. However, the nanoparticles prepared from zirconyl chloride with the subsequent drying of the obtained powder influenced the values τ_10_ and τ_50_ (both for PMDA-ODA and R-BAPS nanocomposites) more pronouncedly compared to the as-prepared nanoparticles (see Table 1). This is also likely explained by the surface features of the nanoparticles, since the drying leads to the removal of OH-groups from the surface and provides better distribution of the filler within a polymer matrix (see Figure 3d,e) and more sites for adsorption of oxygen molecules participating in the formation of the AOS.

Considering nanocomposites containing titania, it is evident that this nanofiller improves their thermal stability. Apparently, it relates to the quite large size (14 nm) of the nanoparticles providing fewer catalytically active centers detrimental for PMDA-ODA matrix, but their amount is still enough for reactions with the sulfonyl radicals in the R-BAPS-based nanocomposites. Moreover, titania nanoparticles are better distributed in the PI matrices (see Figure 2c) and provide substantial enhancement of the mechanical properties (discussed below in Section 3.3) of the films. The nanoparticles are assumed to act as linkers between the polymer macromolecules, thereby inhibiting the thermal decomposition of the materials.

### 3.3. Mechanical Properties

**Figure 7:**
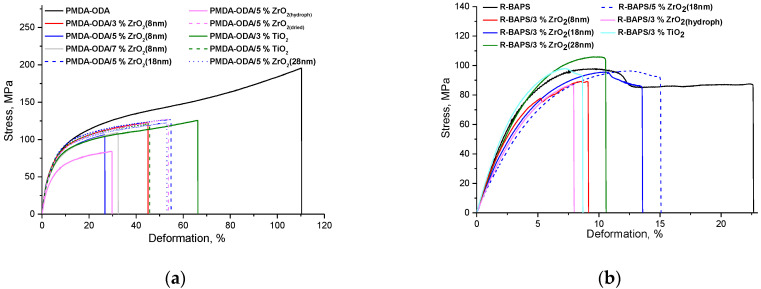
Stress-strain curves of the samples: (**a**) PMDA-ODA-based compositions; (**b**) R-BAPS-based compositions.

The mechanical properties of the PI-based nanocomposites were compared with those of the pristine PI matrices and summarized in Table 2. The stress-strain curve of the PMDA-ODA matrix film is given in Figure 7a. The film stretched uniformly, showing no localized deformation. In contrast, a well-defined maximum indicating the yield point of R-BAPS is clearly evident from its deformation curve (Figure 7b). Such a feature is typical of flexible PIs containing several bridge groups [35], a so-called “neck” being formed on a film. The growth of the “neck” throughout a sample is responsible for its deformation beyond the yield point.

Tensile properties of the nanocomposite PMDA-ODA- as well as R-BAPS-based films exhibit quite similar trends (see Table 2). For instance, at definite combinations of size and concentration, the MO nanofillers augmented the Young’s modulus of both PIs, the yield point also being higher or at least unchanged compared to that of the pristine polymers. However, the ultimate elongation of the composite films always decreased when the nanoparticles were added to the pristine PIs. Such an impact of the nanoparticles on the mechanical properties of not only PIs but also other polymers is quite consistent with the literature data [20,23,36]. The reinforcing effect of MO nanoparticles has been attributed to a certain degree of linking between PI macromolecules and the nanofillers ensuring that external loading is transferred from the continuous polymer phase to the filler. The positive impact of titania was observed to be more pronounced against that of zirconia. Taking into account the SEM images (Figure 2) of the nanocomposites, one could relate this to the less pronounced aggregation and more homogeneous distribution of the former nanoparticles in the PI matrix. These factors along with compatibility of components and their structure are known to be crucial to mechanical characteristics of nanocomposite materials [2].

It is also evident from Table 2 that an increase in the content of ZrO_2_ nanoparticles having the same size led to deterioration in the mechanical characteristics of both PIs (compare the values of Youngs moduli of the PMDA-ODA-based nanocomposites with 3 and 7 wt.% of ZrO_2_(8 nm) and those of the R-BAPS-nanocomposites with 3 and 5 wt.% of ZrO_2_(18 nm)). Obviously, the more nanoparticles are introduced into a PI matrix, the more aggregates, i.e., defects, they form.

Comparing the stiffness and yield point of the nanocomposite materials containing the same amount of zirconia, one may notice that doping both matrix PIs with the larger nanoparticles (either 18 or 28 nm instead of 8 nm) resulted in an enhancement of their mechanical properties (Table 2). This is probably connected with the tendency of the smallest nanoparticles possessing the highest surface energy to agglomerate [33] and to act as stress concentrators, while bigger nanoparticles provide better distribution of the nanofiller within the polymer matrix. This corresponds well with AFM results (compare Figure 3b,c).

It should be pointed out that mechanical characteristics of the nanocomposite materials are strongly affected by the preparation method of the nanoparticles. According to Table 2, the materials containing zirconia nanoparticles obtained from inorganic zirconyl chloride ZrO_2(hydroph)_ have their stiffness and strength drastically deteriorated if the as-prepared zirconia powder was not additionally dried. It supposedly related to the presence of OH-groups on the zirconia surface (Figure S1). On the one hand, these groups can take part in the hydrogen bonding with imidic C=O groups in PIs improving compatibility with the polymer matrix and thereby enhancing mechanical properties of nanocomposite material [37]. On the other hand, the presence of active hydroxyl groups on the surface of the nanoparticles can cause their aggregation [38] in the PAA solution, the aggregates acting as defects in the resulting PI films (compare Figure 3d,e). Moreover, OH-groups may well facilitate adsorption of water molecules on the nanoparticles’ surface thereby damaging the PAA because of the hydrolysis of the acid [39,40]. In contrast, nanocomposites (based on both PMDA-ODA or R-BAPS) filled with preliminarily dried ZrO_2(hydroph)_ nanoparticles turned out to exhibit enhanced mechanical properties.

### 3.4. Thermomechanical Properties

**Figure 8:**
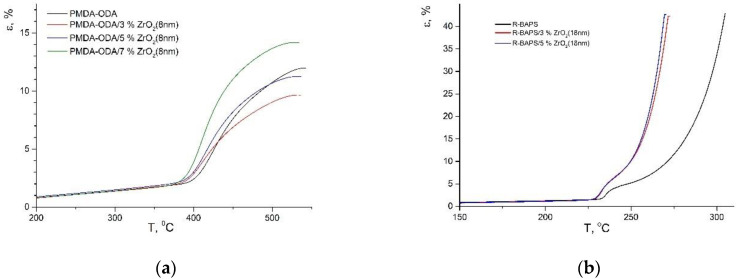
TMA curves of (**a**) PMDA-ODA-based samples and (**b**) R-BAPS-based samples.

In order to investigate mechanical behavior of the nanocomposites while temperature increases, TMA was carried out. It is noteworthy that the two PI matrices under study differ in their thermomechanical performance (see Figure 8), therefore various stress values were applied to the samples in TMA. In the case of semi-flexible PMDA-ODA, and the corresponding nanocomposites, a standard 0.5 MPa loading was used. In flexible R-BAPS, on the other hand, a quite steep transition to a plastic state occurred even under little stress (see Figure 8b), the polymer stretching far beyond the deformation range registered by the setup. Thus, a smaller value of stress, viz. 25 kPa, was applied to R-BAPS-based materials. The analysis was performed in an inert atmosphere so as to prevent the formation of the so-called destruction crosslinks [41,42] between PI macromolecules caused by thermo-oxidative destruction of a polymer (for example, PDMA-ODA begins to decompose in air rather close to *T*_g_). Such crosslinks retard polymer chain mobility after glass transition, thereby complicating the determination of *T*_g_.

The TMA curves of PDMA-ODA-based samples are shown in Figure 8a. The unfilled PI film demonstrated a noticeable deformation above *T*_g_. When the temperature reached 440 °C, an initial stage of the polymer thermal degradation accompanied with the formation of destruction crosslinks might have taken place since the elongation rate decreased gradually. To assess the crosslinking degree, a value of deformation, Δε, in the temperature range between *T*_g_ and a temperature of a maximum deformation on the TMA curve was determined (Table 3).

The effect of different types of nanofillers on the thermomechanical behavior of PMDA-ODA has been determined. It is evident from Figure 3 and Table 3 that regardless of their type, size, and concentration, all the nanoparticles caused a decrease in *T*_g_ of this PI. Since in some cases the stiffness of PMDA-ODA was augmented by the introduction of MO nanoparticles at room temperature (see Section 3.3, Table 2), implying that the nanofiller can act as a linker between the polymer macromolecules, one may suppose that the links may well weaken at higher temperatures (near *T*_g_) or be more flexible compared to the polymer–polymer bonds. Moreover, the nanoparticles and their aggregates may become a hindrance to the inter- as well as intramolecular interactions between PMDA-ODA macrochains, thereby decreasing its *T*_g_.

The introduction of MO nanoparticles also affected the shape of TMA curve of PMDA-ODA at temperatures above *T*_g_, the thermomechanical behavior of the PI depending on the size and concentration of the nanofiller. At the smallest content of ZrO_2_(8 nm), the nanoparticles were supposed to have an impact on segmental mobility of the PI macromolecules, decreasing its flexibility in this temperature range. We have already observed the similar respond of this PI on the introduction of nanoparticles of other types (CeO_2_ and nanocarbon) [35]. Further investigations are needed in order to elucidate the reasons for such an effect. From the practical viewpoint, this means that in the case of the applied mechanical loading not being very high, the polymer can maintain quite high stiffness even above its *T*_g_, which is likely to widen the temperature range of its applicability. The higher the concentration of ZrO_2_(8 nm) nanoparticles, i.e., the more aggregates preventing polymer–polymer interactions are formed, the more flexible behavior PMDA-ODA demonstrates after glass transition. The same corresponds to an increase in size of the nanoparticles from 8 to 28 nm at their constant concentration (5 wt.%) (see Table 3). Considering titania-containing nanocomposites, one should mention that according to mechanical tests (see Section 3.3) and SEM images (Figure 2c), these nanoparticles are better distributed within the polymer matrix and taking part in crosslinking, they can hamper the motion of macromolecular segments more pronouncedly.

As said above, the thermomechanical behavior of R-BAPS and its nanocomposites differs considerably from that of the PMDA-ODA-based samples, the TMA curves of the former having three regions (see Figure 8b). The first one is attributed to a glassy state of the polymer and characterized by small deformations. Due to a number of bridge groups between aromatic rings in R-BAPS macromolecules endowing them with high flexibility, *T*_g_ of this PI is much lower than that of PMDA-ODA (see Table 3 and Table 4). When temperature surpasses *T*_g_, R-BAPS reaches an elastic state. The elastic deformations are superposed by the plastic ones when temperature increases further, and the polymer begins flowing at temperatures ca. 50 °C above the *T*_g_, since the mutual motion of the polymer chains as a whole is eased. The same behavior is typical of other PIs containing several bridge groups [35,43]. Analyzing Table 4, one may notice that MO nanoparticles affect *T*_g_ of R-BAPS only slightly. However, *T*_fl_ (T at which the PI starts flowing) drops by 25–28 °C. A plausible explanation for such a palpable decrease could be that the quasi-spherical MO nanoparticles facilitate a relative motion of the PI macromolecular chains hindering polymer–polymer interactions, the polymer–nanoparticle crosslinked networks responsible for stiffening of the PI nanocomposites at room temperature (see Table 2) being broken by high temperatures. An analogous effect of MO nanoparticles on *T*_fl_ of another PI was registered in [35]. From a practical perspective, such an effect on the PI’s compliance above *T*_g_ can be used in order to reduce the temperature of molding of some PI.

Additionally, the *T*_g_ for the flexible chain R-BAPS-based samples were determined with DTA (see Table 4). The values of *T*_g_ measured by this method turned out to be lower than those registered by the TMA method; however, the trends in the change of this value when comparing the initial PI to the nanocomposites were the same. Moreover, no endothermic peaks indicating the melting of the crystalline phase were registered in the DTA curves indicating that the PI amorphous structure remains upon addition of the MO nanoparticles.

## 4. Conclusions

PI/MO nanocomposites based on PMDA-ODA and R-BAPS polymer matrices and either TiO_2_ or ZrO_2_ nanoparticles were fabricated. SEM and AFM analyses showed that the nanoparticles along with their microaggregates were homogeneously distributed in the PI matrices.

It has been found out that the stability with regard to thermal oxidation of the nanocomposites depends not only on the type and characteristics of the MO nanoparticles, but is also determined by the structure of the matrix PI.

Data were obtained on the effect of the characteristics of MO nanoparticles on the mechanical properties of PIs. It was shown that the introduction of the nanoparticles of a certain size and concentration can cause a significant increase in the rigidity and strength of the resulting nanocomposite materials

The values of *T*_g_ in the unfilled and nanocomposite film samples were determined. It was found that, regardless of their type, size, and concentration, MO nanoparticles caused a decrease in *T*_g_ of both matrix PIs. The effect of MO nanoparticles on the behavior of PI at temperatures above *T*_g_ is demonstrated. Introduction of MO nanofiller into the semiflexible PMDA-ODA can lead to an increase in the rigidity of PI beyond glass transition, which can potentially expand the temperature range of its applicability. In the case of flexible R-BAPS, the introduction of MO nanoparticles caused a significant decrease in *T*_fl_ of materials. Such an effect can be used in reducing the molding temperature of the PIs with a similar structure.

The data presented in this work can be useful for materials science and engineering since they provide the opportunity for the manipulating the functional properties of polyimide nanocomposites by varying the polymer composition as well as the concentration, size, and functionality of MO nanoparticles. This would allow for the design of PI-based materials with desirable functional properties, expanding the scope of their applications.

## Figures and Tables

**Table 1:** Thermal stability indices of polyimides (PIs) and PI-based nanocomposites.

Sample	τ_10_, °C	τ_5__0_, °C
PMDA-ODA	546	578
PMDA-ODA/3 wt.%ZrO_2_(8 nm)	521	567
PMDA-ODA/5 wt.%ZrO_2_(8 nm)	541	577
PMDA-ODA/7 wt.%ZrO_2_(8 nm)	532	569
PMDA-ODA/5 wt.%ZrO_2_(18 nm)	553	581
PMDA-ODA/5 wt.%ZrO_2_(28 nm)	550	583
PMDA-ODA/5 wt.%ZrO_2(hydroph)_	540	584
PMDA-ODA/5 wt.%ZrO_2(dried)_	535	574
PMDA-ODA/3 wt.%TiO_2_	548	584
PMDA-ODA/5 wt.%TiO_2_	550	589
R-BAPS	504	557
R-BAPS/3 wt.%ZrO_2_(8 nm)	512	575
R-BAPS/3 wt.%ZrO_2_(18 nm)	512	572
R-BAPS/3 wt.%ZrO_2_(28 nm)	507	569
R-BAPS/5 wt.%ZrO_2_(18 nm)	509	568
R-BAPS/3 wt.%ZrO_2(hydroph)_	522	575
R-BAPS/3 wt.%ZrO_2(dried)_	524	585
R-BAPS/3 wt.%TiO_2_	516	582
R-BAPS/5 wt.%TiO_2_	523	583

**Table 2:** Mechanical properties of PMDA-ODA- and R-BAPS-based compositions.

Sample	E, GPa	σ_y_, MPa	σ_b_, MPa	ε_b_, %
PMDA-ODA	2.45 (0.06) *	100 (3)	176 (6)	105 (7)
PMDA-ODA/3 wt.%ZrO_2_(8 nm)	2.63 (0.06)	91 (3)	110 (7)	43 (8)
PMDA-ODA/5 wt.%ZrO_2_(8 nm)	2.47 (0.02)	81 (1)	103 (2)	28 (2)
PMDA-ODA/7 wt.%ZrO_2_(8 nm)	2.44 (0.04)	79 (2)	103 (3)	34 (3)
PMDA-ODA/5 wt.%ZrO_2_(18 nm)	2.73 (0.09)	102 (5)	138 (12)	65 (15)
PMDA-ODA/5 wt.%ZrO_2_(28 nm)	2.67 (0.04)	98 (2)	124 (3)	58 (5)
PMDA-ODA/5 wt.%ZrO_2(hydroph)_	2.02 (0.12)	66 (3)	80 (7)	26 (5)
PMDA-ODA/5 wt.%ZrO_2(dried)_	2.60 (0.06)	97 (2)	115 (7)	48 (7)
PMDA-ODA/3 wt.%TiO_2_	2.72 (0.09)	92 (6)	120 (10)	53 (9)
PMDA-ODA/5 wt.%TiO_2_	2.81 (0.08)	100 (2)	119 (5)	48 (5)
R-BAPS	2.67 (0.06)	96 (7)	N/A	23 (3)
R-BAPS/3 wt.%ZrO_2_(8 nm)	2.45 (0.09)	N/A	N/A	9 (1)
R-BAPS/3 wt.%ZrO_2_(18 nm)	2.67 (0.09)	95 (3)	N/A	14 (5)
R-BAPS/3 wt.%ZrO_2_(28 nm)	2.71 (0.07)	110 (3)	N/A	11 (1)
R-BAPS/5 wt.%ZrO_2_(18 nm)	2.10 (0.10)	97 (1)	N/A	14 (2)
R-BAPS/3 wt.% ZrO_2(hydroph)_	1.90 (0.08)	96 (4)	N/A	12 (1)
R-BAPS/3 wt.%ZrO_2(dried)_	2.88 (0.06)	100 (0.2)	N/A	7 (1)
R-BAPS/3 wt.%TiO_2_	2.90 (0.10)	97 (1)	N/A	8.2 (0.3)

Tensile properties. * Standard deviations (SD) are given in brackets.

**Table 3:** Thermomechanical characteristics of PMDA-ODA-based nanocomposites.

Sample	*T*_g_, °C	Δε (ε_max_ − ε*_T_*_g_) *, %
PMDA-ODA	401	9.89
PMDA-ODA/3 wt.%ZrO_2_(8 nm)	391	7.64
PMDA-ODA/5 wt.%ZrO_2_(8 nm)	393	9.16
PMDA-ODA/7 wt.%ZrO_2_(8 nm)	390	12.30
PMDA-ODA/5 wt.%ZrO_2_(18 nm)	393	12.55
PMDA-ODA/5 wt.%ZrO_2_(28 nm)	393	12.96
PMDA-ODA/3 wt.%TiO_2_	387	7.45
PMDA-ODA/5 wt.%TiO_2_	387	6.21

* ε*_T_*_g_ were determined using intersection points of tangents of straight-line regions of the ε(T) functions. ε_max_ were maximal deformations registered in the experiments.

**Table 4:** Thermomechanical characteristics of R-BAPS-based nanocomposites.

Sample	*T*_g_, °C (TMA)	*T*_fl_, * °C	*T*_g_, °C (DTA)
R-BAPS	232	285	224
R-BAPS/3 wt.%ZrO_2_(8 nm)	229	257	221
R-BAPS/3 wt.%ZrO_2_(18 nm)	230	259	223
R-BAPS/3 wt.%ZrO_2_(28 nm)	229	259	224
R-BAPS/5 wt.%ZrO_2_(18 nm)	229	258	223
R-BAPS/3 wt.%TiO_2_	230	260	223

* *T*_fl_ is the temperature at which a sample starts “flowing”. The values were determined using intersection points of tangents of straight-line regions of the ε(*T*) functions in the high-elastic and plastic flow states.

## Data Availability

Not applicable.

## Supplementary Materials

The following supporting information can be downloaded at: https://www.mdpi.com/article/10.3390/polym14132580/s1, Table S1: A list of the nanocomposite samples based on polyimides filled with metal oxide nanoparticles; Figure S1: IR spectra of initial ZrO_2(hydroph)_ (black) and ZrO_2(dried)_ (red) powders.
