# Supplementary material for: Metal Oxide Nanoparticles: An Effective Tool to Modify the Functional Properties of Thermally Stable Polyimide Films
Source: Polymers (Basel). 2022 Jun 25;14(13):2580. doi: 10.3390/polym14132580 (PMC9269602; doi:10.3390/polym14132580)
Supplement: Supplementary file 1 [file polymers-14-02580-s001.zip › Supplementary Table S1.pdf]

Table S1. A list of the nanocomposite samples based on polyimides filled with metal oxide nanoparticles.

Sample	PI	MO	Weight content of nanofiller, wt.%
PMDA-ODA	PMDA-ODA	-	0
PMDA-ODA/3wt.%ZrO_2(8nm)		ZrO_2	3
PMDA-ODA/5wt.%ZrO_2(8nm)			5
PMDA-ODA/7wt.%ZrO_2(8nm)			7
PMDA-ODA/5wt.%ZrO_2(18nm)			5
PMDA-ODA/5wt.%ZrO_2(28nm)			
PMDA-ODA/5wt.%$ZrO_{2(hydroph)}$			
PMDA-ODA/5wt.%$ZrO_{2(dried)}$			
PMDA-ODA/3wt.%TiO_2		TiO_2	3
PMDA-ODA/5wt.%TiO_2			5
R-BAPS	R-BAPS	-	0
R-BAPS/3wt.%ZrO_2(8nm)		ZrO_2	3
R-BAPS/3wt.%ZrO_2(18nm)			
R-BAPS/3wt.%ZrO_2(28nm)			
R-BAPS/5wt.%ZrO_2(18nm)			5
R-BAPS/3wt.% $ZrO_{2(hydroph)}$			3
R-BAPS/3wt.%$ZrO_{2(dried)}$			
R-BAPS/3wt.%TiO_2		TiO_2	
R-BAPS/5wt.%TiO_2			5
