# Supplementary figures and images for: Metal Oxide Nanoparticles: An Effective Tool to Modify the Functional Properties of Thermally Stable Polyimide Films
Source: Polymers (Basel). 2022 Jun 25;14(13):2580. doi: 10.3390/polym14132580 (PMC9269602; doi:10.3390/polym14132580)

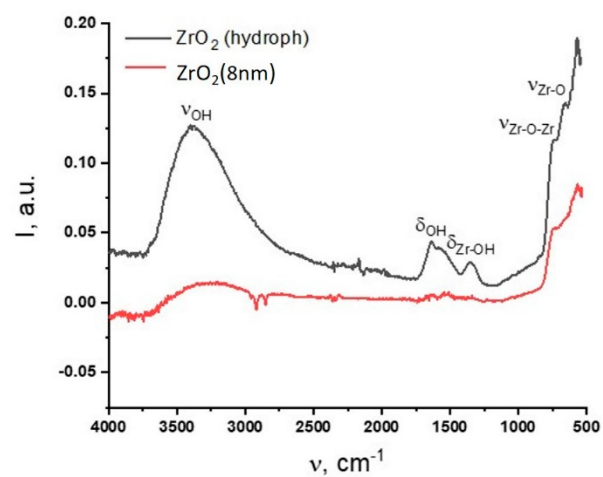

**Figure S1.** IR spectra of initial  $\text{ZrO}_2$ (hydroph) (**black**) and  $\text{ZrO}_2$ (8nm) (**red**) powders.

Supplement: Supplementary file 1 [file polymers-14-02580-s001.zip › Supplementary Figure S1.pdf]
